# Unraveling the future of genomics: CRISPR, single-cell omics, and the applications in cancer and immunology

**DOI:** 10.3389/fgeed.2025.1565387

**Published:** 2025-04-11

**Authors:** A. Vipin Menon, Bicna Song, Lumen Chao, Diksha Sriram, Pamela Chansky, Ishnoor Bakshi, Jane Ulianova, Wei Li

**Affiliations:** ^1^ Center for Genetic Medicine Research, Children’s National Hospital, Washington, DC, DC, United States; ^2^ Department of Genomics and Precision Medicine, George Washington University, Washington, DC, DC, United States; ^3^ The George Washington University, Washington, DC, DC, United States; ^4^ Integrated Biomedical Sciences (IBS) Program, The George Washington University, Washington, DC, DC, United States

**Keywords:** Cas9, Perturb-seq, single-cell CRISPR screen, machine learning, deep learning, therapeutics, clinical research

## Abstract

The CRISPR system has transformed many research areas, including cancer and immunology, by providing a simple yet effective genome editing system. Its simplicity has facilitated large-scale experiments to assess gene functionality across diverse biological contexts, generating extensive datasets that boosted the development of computational methods and machine learning/artificial intelligence applications. Integrating CRISPR with single-cell technologies has further advanced our understanding of genome function and its role in many biological processes, providing unprecedented insights into human biology and disease mechanisms. This powerful combination has accelerated AI-driven analyses, enhancing disease diagnostics, risk prediction, and therapeutic innovations. This review provides a comprehensive overview of CRISPR-based genome editing systems, highlighting their advancements, current progress, challenges, and future opportunities, especially in cancer and immunology.

## 1 Introduction

**FIGURE 1:**
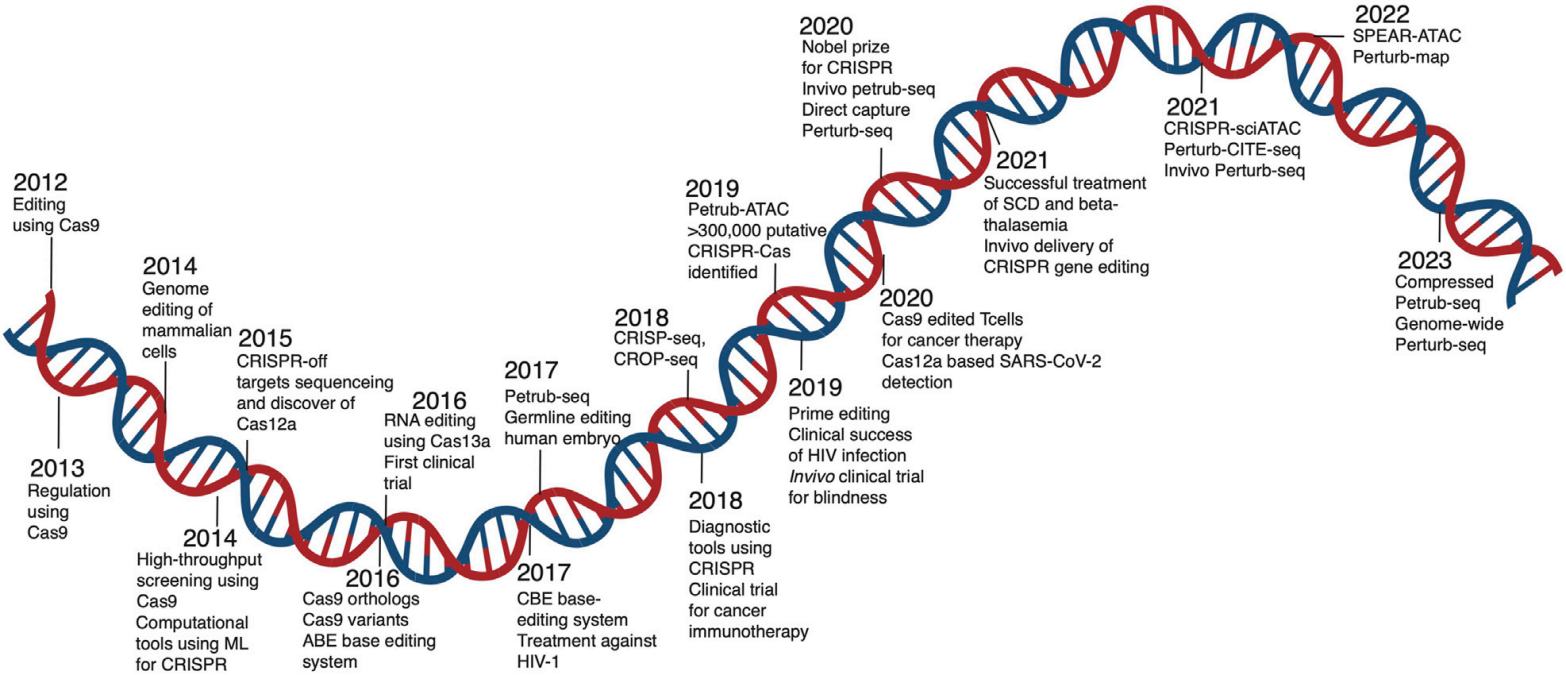
Timeline of CRISPR technology advancement. A brief overview of the development of the CRISPR-Cas tool from research to therapeutic application. The figure showcases advancements in CRISPR technology, application, and integration of single cell screens to enhance biological knowledge.

CRISPR technology has revolutionized genome editing by enabling precise modifications to the genome, resulting in insertions, deletions, or base substitutions (Jiang et al., 2020; Manghwar et al., 2019). Initially discovered as a bacterial immune mechanism (Horvath and Barrangou, 2010), its potential was realized when scientists demonstrated its programmable nature for editing eukaryotic genomes (Jinek et al., 2012). The simplicity of generating targeted edits catalyzed the development of advanced genome editing tools, including CRISPR-Cas9 knockouts (Savic and Schwank, 2016), epigenome editing (Braun et al., 2017; Cervantes-Gracia et al., 2021; Gilbert et al., 2013; Konermann et al., 2015), base/prime editing (Anzalone et al., 2020; Anzalone et al., 2019; Gaudelli et al., 2017), and RNA editing (Abudayyeh et al., 2017; Cox et al., 2017; Xu et al., 2021b; Yu et al., 2024), which have been extensively applied in functional genomics (Przybyla and Gilbert, 2022), therapeutic discovery (Chavez et al., 2023) and disease modeling (Gopal et al., 2020) as shown in Figure 1.

Simultaneously, the emergence of single-cell technologies has revolutionized our understanding of cellular heterogeneity, revealing previously unknown cell states and dynamics. Single-cell RNA sequencing (scRNA-seq) was instrumental in profiling gene expression at the single-cell level, revealing cellular composition and function (Jovic et al., 2022). Advances in multi-omics technologies, such as the integration of scRNA-seq with single-cell ATAC-seq (scATAC-seq) (Jansen et al., 2019) or CITE-seq (Stoeckius et al., 2017), have further refined our ability to map transcriptomic, epigenetic, and proteomic landscapes, enabling the discovery of novel gene regulatory networks (Badia et al., 2023).

The convergence of CRISPR technology with single-cell platforms provides a unique opportunity to investigate gene function and perturbation effects at an unprecedented resolution. CRISPR pooled screens integrated with single-cell readouts enable the identification of gene regulatory networks and cellular responses (Datlinger et al., 2017). Computational approaches have been pivotal in enhancing the precision and interpretability of these studies. For instance, machine learning models have optimized on-target and off-target specificity for CRISPR applications (Chuai et al., 2018), while perturbation scores derived from scRNA-seq data offer quantitative insights into gene functionality (Rood et al., 2024; Song et al., 2023).

The application of these integrated technologies has been particularly impactful in advancing cancer research and immunotherapy. CRISPR-mediated editing has enhanced the efficacy and safety of CAR-T cell therapies, addressing key clinical challenges such as minimizing off-target effects, including cytokine release syndrome (Razeghian et al., 2021). Additionally, multiplex genome editing has allowed for the modification of endogenous T-cell receptors, improving their ability to target and overcome hostile tumor microenvironments (Legut et al., 2018). Beyond oncology, CRISPR has facilitated the development of viral vaccines (Bhujbal et al., 2022) and the engineering of immune cells for personalized medicine, broadening its therapeutic potential and clinical applications.

This review explores the recent advancements in CRISPR technologies and single-cell platforms, emphasizing the critical role of computational tools in bridging experimental data with actionable biological insights. The subsequent sections provide a comprehensive exposition: Section 2 presents a historical and mechanistic overview of the CRISPR-Cas system; Section 3 critically evaluates contemporary technological innovations encompassing targeted gene disruption, epigenetic modulation, and precision base/prime editing methodologies; Section 4 details the integration of CRISPR perturbation screens with single-cell transcriptomic and epigenomic assays to elucidate cellular heterogeneity and complex gene regulatory networks; and Section 5 explores the translational implications of these methodologies in oncology and immunotherapy. By integrating these methodologies, we aim to highlight their transformative impact on functional genomics, therapeutic discovery, and immunotherapy alongside the challenges and opportunities that lie ahead.

## 2 Overview of CRISPR-Cas system

CRISPR-Cas systems are adaptive immune mechanisms in bacteria and archaea that defend against invading genetic elements. These systems consist of CRISPR repeat-spacer arrays, transcribed into CRISPR RNA (crRNA) and trans-activating CRISPR RNA (tracrRNA), along with Cas proteins possessing endonuclease activity (Koonin and Makarova, 2009). CRISPR-Cas systems are categorized into two classes: Class 1 systems involve multi-Cas protein effector complexes (Types I, III, and IV), while Class 2 systems utilize single-effector proteins (Types II, V, and VI) (Hoffmann et al., 2016; Koonin et al., 2017).

The Type II CRISPR-Cas9 system, derived from *Streptococcus pyogenes* (SpCas9), was the first to be characterized and widely applied in genome editing. Cas9, guided by a single-guide RNA (sgRNA)—a fusion of crRNA and tracRNA—recognizes a specific DNA target via the protospacer adjacent motif (PAM) and introduces double-strand breaks (DSBs) (Jinek et al., 2012).

The DSBs generated by Cas9 are repaired through either non-homologous end joining (NHEJ) or homology-directed repair (HDR) pathways (Ceccaldi et al., 2016). NHEJ, the predominant repair mechanism, introduces random insertions or deletions (indels), often resulting in frameshift mutations that inactivate target genes. NHEJ is more efficient in mammals than HDR, as it operates throughout most of the cell cycle and does not require a homologous template (Lieber, 2010; Weterings and Chen, 2008).

## 3 Advancement in CRISPR-Cas system

### 3.1 Evolution of the CRISPR-Cas toolkit

The success of pooled and indel-based screening, combined with SpCas9’s limitations, such as constrained targeting space and off-target effects—has driven the development of Cas9 variants (Chen et al., 2017; Ikeda et al., 2019; Kleinstiver et al., 2015; Lee et al., 2018; Walton et al., 2020). These variants can be broadly categorized into canonical and non-canonical types.

Canonical variants involve mutations in one of the Cas9 endonuclease domains (HNH or RuvC), enhancing editing efficiency while reducing off-target effects (Ikeda et al., 2019; Kim et al., 2020; Kleinstiver et al., 2015; Lee et al., 2018).

In contrast, non-canonical variants feature mutations in the PI domain, expanding the targeting range by relaxing PAM recognition requirements but often at the cost of reduced editing efficiency (Casini et al., 2018; Hu et al., 2018; Kim et al., 2020; Walton et al., 2020).

**FIGURE 2:**
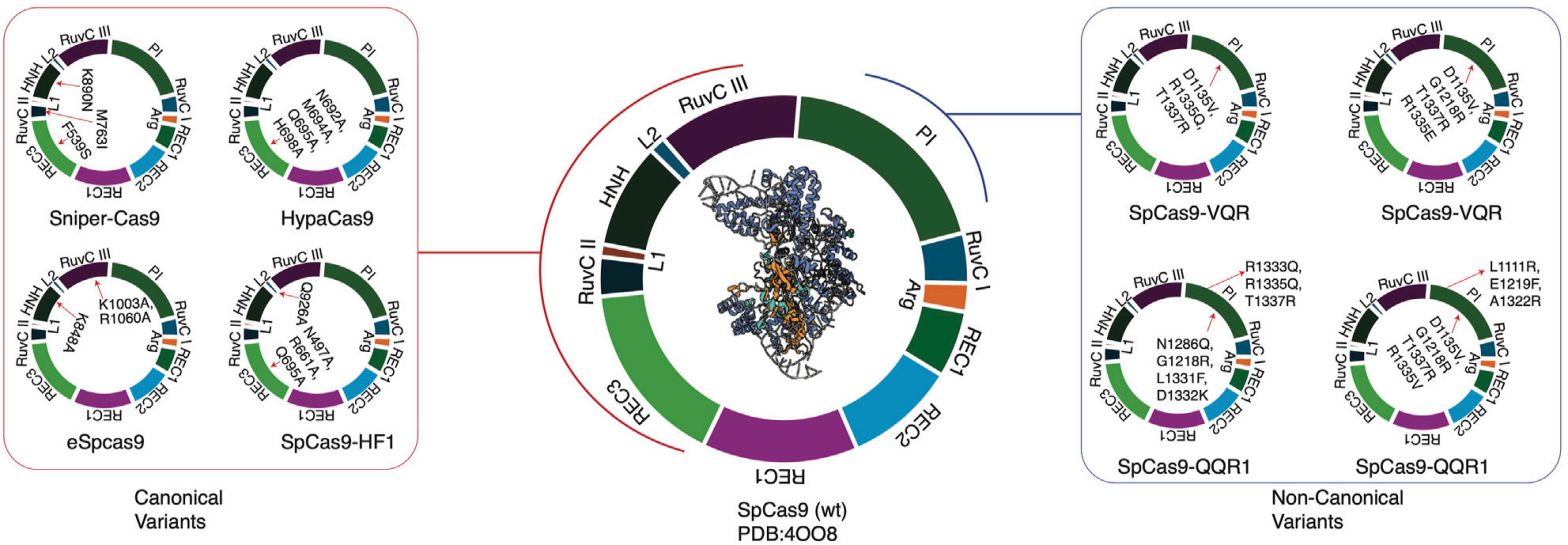
CRISPR variants of SpCas9, along with mutation regions. Non-canonical variants distinctly have PAM interacting (PI) mutations, whereas canonical variants mostly have endonuclease domains or nearby chains.

Canonical variants’ improved performance stems from minimizing non-specific interactions between the HNH domain and target DNA, reducing mismatch tolerance (Chen et al., 2017). Non-canonical variants significantly enhance targeting flexibility by relaxing the PAM-PI complex (Nishimasu et al., 2018) (Figure 2).

The availability of these Cas9 variants, coupled with high-throughput screening techniques, has dramatically expanded the CRISPR-Cas toolkit. This versatility empowers researchers to tailor CRISPR systems to specific experimental requirements, advancing functional genomics, therapeutic discovery, and beyond (Supplementary Table 1).

### 3.2 CRISPR-Cas in gene regulation and therapeutics

The Cas9 system and its variants were initially employed for functional gene assessment through gene knockout. However, to investigate the roles of transcripts and epigenetics in cellular state development, Cas9 underwent significant protein engineering. Early efforts focused on mutating the catalytic domains of Cas9—RuvC and HNH—to render them catalytically inactive, resulting in nuclease-dead Cas9 (dCas9). This dCas9 was repurposed for transcriptional regulation by fusing it with effector domains (Gilbert et al., 2014). For transcriptional repression, dCas9 was fused to the KRAB (Krüppel-associated box) domain [CRISPRi (Bikard et al., 2013; Gilbert et al., 2014; Gilbert et al., 2013)]. In contrast, transcriptional activation was achieved by coupling dCas9 with the MS2-VP16 hybrid protein [CRISPRa (Gilbert et al., 2014; Gilbert et al., 2013; Konermann et al., 2015)]. These modifications were pivotal in advancing research across human and mouse models and mouse embryos, enabling the identification of key regulatory genes critical for cellular development and gene regulatory networks (Kearns et al., 2014).

However, incorporating repressor or activator domains into the dCas9-sgRNA-target complex introduces additional layers of structural complexity to the CRISPRi/a system. This increased complexity results in a more rigid structural conformation, limiting the system’s flexibility and efficiency. Furthermore, structural rigidity can influence the spatial orientation of the complex, impacting its ability to function optimally at target loci. Additionally, the intricate structure of the CRISPRi/a system can inadvertently affect the expression of neighboring genes, leading to a loss of precise control and an increase in off-target effects. Despite its potential, the modifications introduced by CRISPRi/a remain temporary and limited in scale. These limitations have restricted its broader clinical and therapeutic applications, underscoring the need to develop more robust and scalable systems to enable effective clinical translation.

Transiting gene editing tools to clinical or therapeutic relevance necessitates minimal off-target effects, precise editing, and efficient delivery. To address these challenges, base and prime editors were developed, enabling precise point mutations without requiring double-strand breaks (DSBs) or donor templates (Gaudelli et al., 2017; Komor et al., 2017; Molla and Yang, 2019; Yang et al., 2019; Komor et al., 2016; Anzalone et al., 2019). These advancements marked a significant step forward in enhancing the precision and applicability of CRISPR-based systems.

The base editor system comprises catalytically impaired Cas9, sgRNA, and a deaminase enzyme. Depending on the deaminase, base editors are classified into Cytosine Base Editors (CBEs) and Adenine Base Editors (ABEs). CBEs use cytidine deaminase to convert C•G base pairs to T•A base pairs (Komor et al., 2016), while ABEs use deoxyadenosine deaminase to catalyze A•T-to-G•C conversions (Gaudelli et al., 2017). Both ABEs and CBEs mediate all four possible transition mutations (C→T, A→G, T→C, G→A), which collectively account for 30% of currently annotated human pathogenic variants (Landrum et al., 2016). These systems have demonstrated therapeutic potential, such as correcting pathogenic mutations in the HBB promoter by converting C•G to T•A in the BCL11A erythroid enhancer, a strategy to address hemoglobinopathies (Zeng et al., 2020). Additionally, advanced base editor systems [e.g., BE4 and higher (Koblan et al., 2018)] have incorporated high-fidelity Cas9 variants to enhance targeting scope and reduce both gRNA-dependent and independent off-target effects (Anzalone et al., 2020; Rees et al., 2017; Xu et al., 2019), thereby broadening their utility in therapeutic applications.

Despite these advancements, base editors have limitations. They are unable to mediate transversion mutations (e.g., C•G-to-A•T, C•G-to-G•C, T•A-to-A•T, T•A-to-G•C), and they cannot introduce insertions or deletions. Additionally, undesired bystander mutations may occur when multiple target nucleotides fall within the base editing window. To overcome these constraints, prime editors (Anzalone et al., 2020; Rees and Liu, 2018) were developed (Anzalone et al., 2019), enabling all 12 possible types of point mutations and small insertions and deletions with favorable editing-to-indel ratios.

The prime editing system employs fusion proteins comprising a Cas9 nickase domain (inactivated HNH nuclease) and an engineered reverse transcriptase. The system is guided by a prime editing guide RNA (pegRNA), which specifies the target site through its spacer sequence and encodes the desired edit within a 3′extension of the pegRNA (Anzalone et al., 2019). Prime editors have been successfully tested in multiple human cell lines (Anzalone et al., 2019), postmitotic mouse cortical neurons (Anzalone et al., 2019), human induced pluripotent stem cells (Surun et al., 2020), and mouse embryos (Liu Y. et al., 2020). However, their application in clinical and therapeutic contexts remains limited compared to base editors. This limitation is primarily due to an incomplete understanding of DNA repair mechanisms underlying productive *versus* unproductive prime edits and challenges in delivering complex pegRNA constructs *in vivo*. Nevertheless, recent advancements, such as shrinking the size of reverse transcriptase and manipulating DNA repair pathways to favor 3′edited flaps over 5′flaps, have shown promise in improving the efficiency and viability of prime editing systems for clinical use (Anzalone et al., 2020).

Together, base editors and prime editors complement each other, addressing distinct mutational needs and expanding the scope of precise genome editing. These innovations bring us closer to realizing the full potential of CRISPR-based systems for clinical and therapeutic applications, paving the way for gene therapy-driven treatments.

### 3.3 Computational insights and advances in CRISPR-Cas systems

The advent of CRISPR-Cas systems has revolutionized genome and epigenome editing, accelerating advancements in functional genomics. This progress underscores the importance of accurately characterizing and quantifying genome editing outcomes to facilitate the development of novel tools and bridge the knowledge gap between genome sequence and function (Clement et al., 2020). Numerous CRISPR-based screening methods have been developed, including pooled (Shalem et al., 2015; Wang et al., 2014), tiling (He et al., 2019), and indel-focused approaches (Kim et al., 2017), which rely heavily on next-generation sequencing (NGS). These methodologies involve multiple downstream processing steps, necessitating robust computational tools to ensure precise data analysis.

#### 3.3.1 Preprocessing tools for screening datasets

The downstream analysis of CRISPR-based screening data begins with preprocessing steps critical for ensuring data quality and reliability. Key tasks include removing sequencing artifacts, reading alignment to reference genomes, quantifying sgRNA abundance, and normalizing to minimize experimental biases. Computational tools tailored to these tasks streamline the data processing pipeline, enhancing the reproducibility and interpretability of experimental results (Li et al., 2023). Among the most widely adopted tools are **CRISPResso** and **MAGeCK**, each designed for distinct aspects of CRISPR data analysis.

**CRISPResso** (Pinello et al., 2016) is a versatile tool for qualitative and quantitative assessment of genome-editing outcomes at target loci using NGS data. It evaluates sequence quality, ensures high alignment fidelity, and measures insertions, deletions, and nucleotide substitutions with precision. Furthermore, it detects frameshift mutations and quantifies repair outcomes, enabling comprehensive evaluation of editing accuracy. The advanced version, **CRISPResso2** (Clement et al., 2019), extends its capabilities to encompass base and prime editing experiments, support multiple editing types, and perform allele-specific quantification in heterozygous references. This makes CRISPResso an indispensable tool for studies requiring precise characterization of genomic alterations.

On the other hand, **MAGeCK** [Model-based Analysis of Genome-wide CRISPR/Cas9 Knockout (Li et al., 2014)] is tailored for large-scale pooled CRISPR screening experiments. It excels in identifying positively and negatively selected genes under different experimental conditions, providing critical insights into gene functions and pathways. MAGeCK then employs a negative binomial (NB) model to assess significant differences in sgRNA abundance between treatment and control groups. Using the Robust Ranking Algorithm [RRA (Kolde et al., 2012)], MAGeCK prioritizes genes and pathways, offering key insights into gene functions and regulatory networks.

Together, CRISPResso and MAGeCK address complementary aspects of CRISPR-based data analysis. CRISPResso focuses on the high-resolution characterization of editing events, while MAGeCK enables genome-wide functional genomics exploration. Beyond CRISPResso and MAGeCK, numerous additional tools are available for the preprocessing and analysis of CRISPR-based screening datasets, as detailed in Supplementary Table 2. These resources offer researchers diverse options for tailoring their workflows to maximize the utility and interpretability of CRISPR screening experiments, fostering advancements in genome editing and functional genomics.

#### 3.3.2 sgRNA design for on and off-target CRISPR activity

The downstream analysis of CRISPR-based experiments has provided critical insights into editing events and their impact on genomic functions. However, the success of these experiments fundamentally depends on the efficiency and specificity of the single guide RNA (sgRNA) or prime editing guide RNA (pegRNA) sequence, which directs the Cas enzyme to the target site for editing or substitution. Efficiency reflects the sgRNA’s ability to target specific sequences effectively, while specificity determines whether the editing events are unique to the intended site or result in unintended off-target effects. Several factors influencing efficiency and specificity have been incorporated into the design principles for sgRNA sequences [(Chuai et al., 2017)]. Below, we discuss the advancements in the design of sgRNAs for on-target and off-target activity.

##### 3.3.2.1 On-target activity design

The on-target activity of sgRNA/pegRNA largely hinges on the nucleotide composition and structural properties of the sgRNA sequence. Foundational studies from the Broad Institute (Doench et al., 2014; Wang et al., 2014) revealed specific nucleotide biases that enhance on-target efficiency. For instance, guanine immediately adjacent to the protospacer adjacent motif (PAM) significantly improves targeting efficiency, whereas cytosine in the same position reduces efficiency. Conversely, in the seed region of the sgRNA, cytosine is favored, while guanine is less preferred. These preferences, validated through statistical binomial tests, have established a predictive framework for designing sgRNAs with optimal on-target performance.

Despite these heuristic approaches, traditional methods often have limited generalizability and adaptability to unseen datasets. Supervised machine learning models were developed to address this, incorporating sgRNA sequence features and biological context to generate predictive scores that rank sgRNAs for potency in diverse datasets. Early machine learning models, such as support vector machines [SVM (Wang et al., 2014)], linear regression (Xu et al., 2015), and logistic regression (Menon et al., 2020), utilized sgRNA sequences encoded in a one-hot format alongside continuous biological features such as GC content, self-folding energy, and melting temperature. These models laid the foundation for more robust assessments by assigning scores that reflected sgRNA efficacy.

Later advancements integrated ensemble learning methods, including gradient boosting (Doench et al., 2016) and random forests (Rahman and Rahman, 2017), to better handle data complexity and improve predictive robustness. These algorithms enhanced performance in ranking sgRNAs by incorporating diverse datasets and capturing intricate sequence-function relationships.

Deep learning models emerged as transformative tools for sgRNA optimization as the on-target experimental designs evolved. Frameworks such as convolutional neural networks [CNNs (Kim et al., 2019; Kim et al., 2018; Kim et al., 2024; Kim et al., 2020; Yu et al., 2023)] leverage weight-sharing strategies to capture hierarchical spatial patterns in input sequences, while Long Short-Term Memory [LSTM (Wang et al., 2019)] networks excel in modeling dynamic sequence information. With their ability to abstract k-mer-based features (Akay et al., 2024), transformers have been integrated with CNNs to yield interpretable, high-precision results (Liu et al., 2019). **CHOP-CHOP** (Labun et al., 2019) and **CRISPOR** (Concordet and Haeussler, 2018) integrate these advanced algorithms to balance on-target efficiency with other considerations, such as GC content and PAM-proximal preferences, allowing researchers to design sgRNAs tailored to their experimental needs. Species-specific platforms like **CRISPR-PLANT** (Xie et al., 2014), **CRISPR-P** (Lei et al., 2014), **DRSC** (Patrick et al., 1988), and **EuPaGDT** (Peng and Tarleton, 2015) extend these capabilities to accommodate unique genomic contexts, such as polyploid species or gene family targeting. These tools ensure high precision in experimental designs by incorporating features relevant to specific organisms.

##### 3.3.2.2 Off-target activity analysis

Complementing the focus on on-target design and assessing off-target activity is equally critical. Next-generation sequencing (NGS)-based methods have been developed to evaluate off-target effects, including GUIDE-seq (Tsai et al., 2015), CIRCLE-seq (Tsai et al., 2017), Digenome-seq (Park et al., 2017), and DISCOVER-seq (Wienert et al., 2019). These methods detect double-strand breaks (DSBs) caused by CRISPR nucleases across the genome, using approaches such as marker integration (GUIDE-seq), *in vitro* DNA digestion (Digenome-seq), circular DNA cleavage (CIRCLE-seq), or repair protein enrichment (DISCOVER-seq). The resulting cleaved DNA fragments are mapped to the genome to identify off-target sites for a given sgRNA sequence.

Early computational tools for analyzing off-target effects, such as CCTop (Stemmer et al., 2015), MIT Score (Haber et al., 2016), CFD Score (Doench et al., 2016), and CropIT (Singh et al., 2015), ranked sgRNAs based on minimal off-target activity. However, these heuristic methods could not often integrate on-target design considerations and struggled to capture complex relationships between on- and off-target sequences.

The introduction of integrated computational tools has addressed these limitations. Platforms like Elevation (Listgarten et al., 2018) leverage gradient boosting to incorporate both on-target and off-target considerations, enabling holistic sgRNA design. Subsequent efforts employed deep learning algorithms to refine off-target analysis. These models process sgRNA-DNA sequence pairs encoded as one-hot representations (Chuai et al., 2018; Lin and Wong, 2018), word embeddings (Liu et al., 2019; Liu Q. et al., 2020; Zhang et al., 2021), or numerical embeddings (Vinodkumar et al., 2021). Supervised learning approaches, particularly classification frameworks, have demonstrated superior efficiency over regression models for off-target prediction.

Modern tools for sgRNA design integrate experimental data with sophisticated computational algorithms to ensure high efficiency and specificity. This empowers researchers to optimize genome editing across various scientific disciplines. These advancements, facilitated by machine learning and deep learning, enable precise and reliable CRISPR-based applications.

As CRISPR applications diversify, these tools and interfaces evolve to support new experimental paradigms, including base editing, prime editing, and RNA targeting. Comprehensive databases, tools, and interfaces tailored for such applications are listed in Supplementary Table 3, providing a curated repository for researchers to explore and adopt cutting-edge CRISPR technologies.

##### 3.3.2.3 Potential advances in AI for designing CRISPR-Cas system

The development of web-based tools and databases has streamlined the design of sgRNAs for CRISPR-Cas systems, emphasizing on-target efficiency and minimizing off-target effects. Integrating artificial intelligence (AI) into CRISPR design builds on these advancements and introduces a transformative layer of precision and innovation. While web-based tools focus on optimizing sgRNA sequences using established algorithms and user-friendly interfaces, recent progress in AI, mainly through **Large Language Models (LLMs)**, extends these capabilities by addressing complex relationships and structural features that were previously challenging to model computationally.

Recent studies underscore the importance of structural features in improving editing efficiency. For instance, systemic modifications to the spacer and scaffold regions of sgRNA or pegRNA have significantly enhanced editing outcomes. A study (Huszar et al., 2023) demonstrated that alterations in the SL1 and SL2 regions of the scaffold improve prime editing efficiency without affecting the stability of the Cas9-pegRNA complex. Furthermore, another study (Li et al., 2022) highlighted that introducing RNA G-quadruplex structures into sgRNA or pegRNA design can boost modification rates by up to 80%. Despite these promising findings, incorporating such intricate features into existing computational frameworks remains a significant challenge due to the limitations of traditional algorithms.

Traditional deep learning models like CNNs and RNNs are primarily designed to capture statistical correlations rather than the mechanistic, biologically meaningful interactions that drive CRISPR efficiency. CNNs, for example, rely on fixed filter sizes to detect local sequence motifs, which can cause them to miss long-range interactions crucial for effective sgRNA targeting. On the other hand, RNNs—even when utilizing variants like LSTM or GRU—can encounter issues such as exploding gradients, which restrict their ability to model extended genomic sequences where distal interactions may play a significant role.

Furthermore, these architectures are inherently focused on statistical association, often leading to predictions that do not necessarily reflect the underlying biological processes. This is particularly limiting when attempting to incorporate advanced structural features into models. Traditional approaches like XGBoost, while effective in handling non-linear relationships, require extensive feature engineering to capture spatial and sequential dependencies, resulting in a loss of nuanced structural information. Consequently, the predictive power of these models may be compromised when dealing with the sophisticated modifications observed in CRISPR systems.

This is where LLMs, a class of AI models initially designed for natural language processing, have shown remarkable promise. These models (Nguyen et al., 2024; Zhou Z. et al., 2023) excel at extracting and analyzing sequence information, outperforming traditional deep-learning models in tasks such as decoding epigenetic patterns, understanding transcriptional regulation, and identifying disease associations. Beyond analysis, LLMs have been adapted to generate customizable gene editors directly derived from Cas operons (Ruffolo et al., 2024). By bypassing evolutionary constraints, these models create gene editors with optimal properties, achieving activity and specificity levels comparable to or surpassing SpCas9. Further expanding the scope of LLM applications, Li et al. (2024) employed a reconfigured protein-based LLM to discover an alignment-free CRISPR-Cas system capable of self-processing pre-crRNA. Their method involved four steps: (1) using a protein LLM to discover Cas homologs, (2) employing the model to facilitate self-processing of pre-crRNA, (3) conducting phylogenetic analysis to identify candidate Cas12 enzymes, and (4) determining the required protospacer adjacent motif (PAM). Experimental validations have confirmed the robustness and reliability of these AI-generated designs, highlighting the transformative potential of LLMs in advancing gene editing technologies. Experimental validations further demonstrate the robustness and reliability of these AI-generated designs.

LLMs’ application extends beyond sequence generation to encompass structural features critical to editing efficiency and specificity. These models have the potential to model interactions such as spacer-scaffold base pairing, the Cas9-sgRNA-target complex, and the incorporation of RNA G-quadruplexes, which are key to enhancing editing precision. By integrating such structural elements, LLMs could redefine the landscape of genome editing, enabling the design of efficient and precise tools.

The advances brought by AI, particularly LLMs, complement the progress made by web-based tools and databases, bridging gaps in CRISPR-Cas design that traditional algorithms could not address. Together, these technologies are poised to propel the field of genome editing to new heights, offering researchers a comprehensive toolkit for designing, optimizing, and implementing CRISPR-based experiments with unprecedented precision and efficiency.

## 4 Convergence of CRISPR and single-cell technologies

### 4.1 High-throughput pooled CRISPR screening

The ability to generate targeted edits, coupled with efficient repair mechanisms, has enabled the development of high-throughput, genome-scale functional screening. This capability, combined with the flexibility of high-throughput screening methodologies, has catalyzed the development of genome-scale pooled CRISPR screening approaches. These screens allow researchers to systematically investigate gene functions, interactions, and pathways in various biological contexts.

Early studies [62, 63] demonstrated the feasibility of this technique using single lentiviral vectors to deliver key components, including Cas9, single-guide RNAs (sgRNAs), and selectable markers, directly into target cells. This streamlined delivery mechanism ensures consistent expression of all necessary components within a single cell, enabling robust and reproducible gene targeting. These vectors often incorporate fluorescent or drug-resistance markers, which facilitate the selection and tracking of successfully transduced cells.

The pooled screening workflow begins with the design and synthesis of a library containing thousands of sgRNAs targeting specific genes of interest or the entire genome. Each sgRNA is linked to a unique barcode, enabling high-throughput analysis. Once delivered into a population of cells, the CRISPR machinery induces gene-specific edits across the genome. By coupling these screens with high-throughput sequencing, researchers can quantitatively assess the abundance of each sgRNA within the population, providing insights into the impact of gene perturbations on cell fitness, survival, or other phenotypic traits.

This approach is compelling for identifying essential genes, drug targets, and genetic modifiers of disease phenotypes (Doench et al., 2016). By simultaneously targeting multiple genes, pooled screens enable the dissection of complex gene networks and pathways, revealing synergistic or antagonistic relationships that would be difficult to uncover using traditional single-gene approaches.

The workflow is highly scalable and is adapted for various applications, including loss-of-function screens using knockout libraries (Doench et al., 2016; Doench et al., 2014; Wang et al., 2014), gain-of-function screens through CRISPR activation (CRISPRa) (Konermann et al., 2015), and epigenetic studies leveraging CRISPR interference (CRISPRi) (Gilbert et al., 2014). This streamlined approach facilitated the simultaneous targeting of multiple genes, enabling a comprehensive analysis of gene functionality (Chari et al., 2015; Wilson et al., 2018).

### 4.2 Single-cell CRISPR screens

**FIGURE 3:**
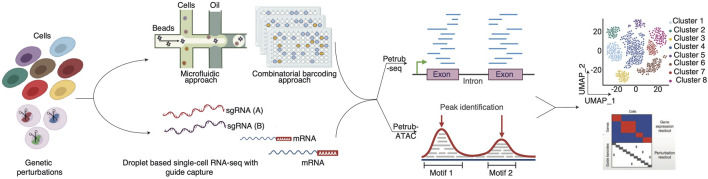
Overview of single-cell CRISPR screen analysis (Petrub-seq and Petrub-ATAC). This figure illustrates the integrated workflow and analytical framework employed to interrogate the effects of CRISPR perturbations at single-cell resolution, utilizing two complementary platforms: Petrub-seq for transcriptomic profiling and Petrub-ATAC for chromatin accessibility mapping.

Integrating pooled screen CRISPR-Cas systems with advanced single-cell technologies represents the next Frontier in genome editing and functional genomics. Building on the precision of CRISPR design tools and the computational advancements discussed earlier, single-cell CRISPR screens combine the targeted genome-editing capabilities of CRISPR with the high-resolution profiling power of single-cell transcriptomics and multiome technologies (Figure 3). This convergence enables researchers to interrogate genetic and molecular mechanisms unprecedentedly, offering more profound insights into cellular behavior and heterogeneity (Ghamsari et al., 2023).

Single-cell RNA sequencing [scRNA-seq (Jaitin et al., 2014)] and multiome technologies, which profile both transcriptomics and chromatin accessibility [e.g., scATAC-seq (Cusanovich et al., 2015)], have transformed the study of cellular diversity and regulatory landscapes. These techniques provide high-resolution snapshots of cellular states, capturing gene expression and epigenetic dynamics in individual cells. For example, scRNA-seq has uncovered transcriptomic diversity across tissues, elucidating the functional roles of distinct cell populations. Multiome technologies, by integrating chromatin accessibility data with transcriptional profiles, further enhance our understanding of gene regulation by linking chromatin states to transcriptional outcomes.

Combined with CRISPR-Cas systems, these single-cell approaches allow researchers to dissect the functional consequences of genetic perturbations at an unparalleled scale. Single-cell CRISPR screens leverage CRISPR-based gene editing to induce targeted perturbations and single-cell technologies to measure the resultant changes in gene expression, chromatin accessibility, or both. This dual capability enables high precision identification of gene regulatory networks, cellular pathways, and context-specific dependencies. For instance, perturbations introduced by CRISPR can now be linked to cellular phenotypes captured through single-cell multi-omic profiling, revealing how genetic modifications shape regulatory landscapes and drive cellular responses.

This convergence of CRISPR and single-cell technologies builds directly upon the foundational advancements in on-target and off-target sgRNA design and optimization discussed earlier. As CRISPR tools become increasingly precise and AI models enhance their predictive capabilities, applying single-cell methodologies to CRISPR screens offers an integrated platform to study complex biological systems at both the genetic and molecular levels. Combining precise genetic perturbations with single-cell resolution readouts, single-cell CRISPR screens provide an unparalleled ability to interrogate how specific genetic changes affect cellular phenotypes. These approaches allow researchers to connect genotype to phenotype with unprecedented granularity, directly associating genetic variations with their functional consequences.

Moreover, these methods address key challenges in understanding cellular heterogeneity. Traditional bulk assays average the differences between individual cells, obscuring critical insights into diverse cellular states. In contrast, single-cell CRISPR screens capture cell variability, revealing how distinct populations respond to genetic perturbations. Incorporating multiome platforms further enhances this capability, providing an integrated view of how gene expression and chromatin accessibility are altered in response to targeted genome edits.

For example, Perturb-seq (Dixit et al., 2016), has been widely used to map the transcriptional consequences of CRISPR-based gene knockouts using scRNA-seq. Similarly, multiome platforms such as Perturb-ATAC (Rubin et al., 2019) link CRISPR perturbations to chromatin accessibility changes, elucidating the regulatory networks that drive cellular processes. These advancements have proven invaluable in studying diverse biological systems, including immune cell activation, differentiation pathways, and cancer progression.

While single-cell CRISPR screens have provided unprecedented resolution in studying genetic perturbations, they do not capture the spatial context in which cells interact and function. However, recent advancements in spatial transcriptomics have enabled the integration of CRISPR-based screening with spatially resolved molecular profiling, allowing researchers to study gene function in a tissue-specific context. Emerging technologies such as Perturb-map (Dhainaut et al., 2022), Perturb-FISH (Binan et al., 2025), Perturb-DBiT (Baysoy et al., 2024), and Perturb-Multi (Saunders et al., 2024) have extended the capabilities of single-cell CRISPR screens by incorporating spatial information, bridging the gap between perturbation-based functional genomics and spatially organized cellular environments. These spatial CRISPR screens provide valuable insights into tissue architecture, cell-cell interactions, and microenvironmental influences on gene regulation, offering a new dimension to functional genomics.

The convergence of genome-editing tools with single-cell and multiome technologies marks a significant leap forward in decoding the genetic underpinnings of cellular behavior. It enables researchers to explore gene regulatory networks, uncover disease mechanisms, and identify potential therapeutic targets with remarkable precision. This integration is poised to shape the future of genomic research by providing deeper insights into the complex interplay between genes and cellular function.

### 4.3 scRNA-seq and CRISPR perturbation to link genotype with phenotype at the cellular level

Building on integrating single-cell technologies with genome-editing tools, the combination of CRISPR perturbations with scRNA-seq represents a powerful approach to uncovering genotype-to-phenotype relationships in diverse cellular contexts. This approach addresses a critical challenge in modern biology: linking specific genetic changes to their phenotype outcomes in heterogeneous cell populations. By enabling precise gene editing and simultaneous profiling of transcriptomic changes at single-cell resolution, these methods have opened new avenues for functional genomics.

The implementation of this strategy relies on platforms like Perturb-seq (Dixit et al., 2016), CROP-seq (Datlinger et al., 2017), and CRISP-seq (Jaitin et al., 2016), each of which exemplifies how CRISPR perturbations can be systematically combined with scRNA-seq to generate detailed functional data (Supplementary Table 4). Perturb-seq, for instance, integrates pooled CRISPR screens with single-cell transcriptional profiling, allowing researchers to assess the effects of multiple gene knockouts within a single experiment. Its use of molecular barcodes enables direct association between specific perturbation and their transcriptional outcomes, offering a high throughput means of dissecting gene regulatory networks. Similarly, CROP-seq simplifies the delivery of guide RNAs by utilizing a lentiviral vector system, making the approach more accessible and scalable for diverse experimental settings. CRISP-seq, as one of the earlier implementations, demonstrated the feasibility of linking genetic perturbations to transcriptomic changes, setting the stage for subsequent advancements in the field.

These technologies have significantly advanced the study of cellular heterogeneity, revealing how individual cells within a population respond differently to genetic perturbations. In immune cells, for example, single-cell CRISPR screens have uncovered key activation and differentiation pathways regulators. At the same time, in cancer models, they have highlighted resistance mechanisms to therapeutic agents. Beyond their utility in identifying gene functions, these approaches provide a framework for mapping gene regulatory networks by integrating perturbation-induced transcriptional changes across diverse cell types.

The versatility of CRISPR perturbation combined with scRNA-seq extends to understanding complex phenotypes in healthy and diseased states. Researchers can use these methods to study developmental processes, such as lineage specification and differentiation, or to investigate how genetic variations contribute to pathological conditions. The ability to systematically analyze perturbations at single-cell resolution has been particularly impactful in studies of an immune response, where it has identified genes driving specific functional states (Zhou P. et al., 2023), and in developmental biology, tracing the progression of distinct cell fates (Yang et al., 2022).

By connecting targeted genetic edits to cellular transcriptional profiles, CRISPR perturbation with scRNA-seq provides a transformative tool for decoding the complexity of biological systems. This approach continues to drive innovations in functional genomics, setting the stage for a more comprehensive exploration of cellular responses to genetic and environmental changes.

### 4.4 Computational technologies for single-cell perturbation

The integration of CRISPR perturbation with scRNA-seq has provided powerful experimental tools to probe genetic functions at a granular level. However, the full potential of these technologies relies on sophisticated computational approaches that can analyze and interpret the vast and complex datasets generated. As single-cell perturbation experiments scale in complexity, computational frameworks have become indispensable for identifying meaningful patterns, quantifying perturbation effects, and linking them to cellular phenotypes.

**FIGURE 4:**
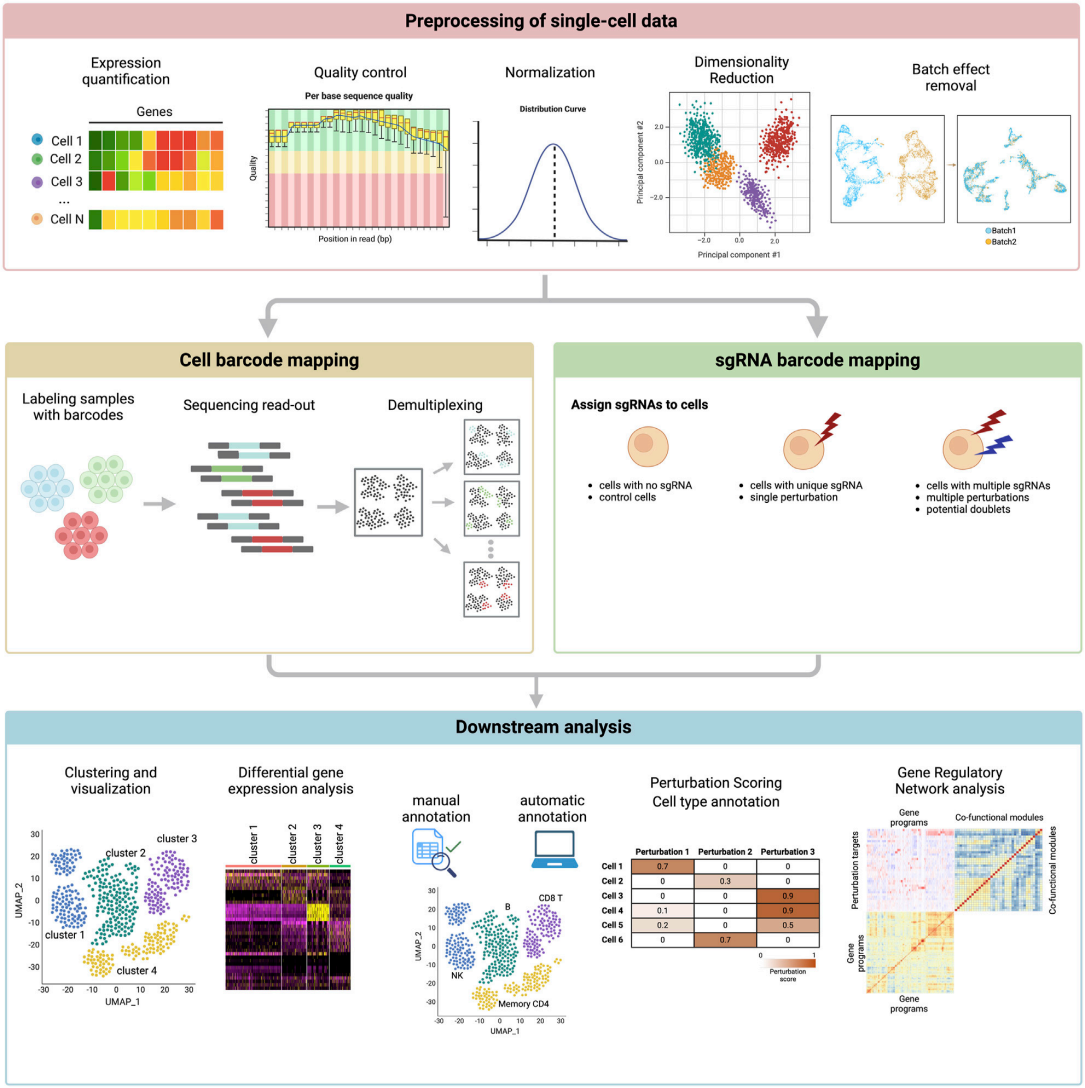
Computational pipeline for single-cell perturbation data. The figure depicts steps in the pipeline that begins with 1) preprocessing, 2) cell and sgRNA barcode mapping, and finally 3) the downstream analysis.

One of the foundational challenges addressed by computational tools is the quantification of perturbation-induced effects across individual cells. Metrics (Rood et al., 2024) such as the perturbation-response score (PS) framework [(Song et al., 2023); Mixscape (Papalexi et al., 2021)] have been developed to measure how much a genetic perturbation influences a cell’s transcriptional state (Figure 4). These approaches use statistical modeling to distinguish genuine biological effects from technical noise, ensuring robust and reproducible insights. As perturbation datasets grow, scalable methods for analyzing millions of cells simultaneously have become critical, enabling researchers to extend these analyses across diverse cell types and experimental conditions.

Computational tools also play a crucial role in integrating single-cell perturbation data with other layers of information, such as chromatin accessibility, protein expression, or metabolic states. Advances in machine learning, including algorithms leveraging variational autoencoders (Lopez et al., 2018) and graph-based approaches (Roohani et al., 2024; Wolf et al., 2018), have allowed for dimensionality reduction, feature selection, and cell clustering, all essential for uncovering the functional consequences of genetic perturbations. These methods enable researchers to deconvolve complex datasets and focus on the key regulatory relationships that drive cellular behavior.

Another significant computational advancement has addressed batch effects and noise inherent in single-cell experiments. Tools such as Harmony (Korsunsky et al., 2019), LIGER (Welch et al., 2019), and, more recently, deep learning-based frameworks (Lotfollahi et al., 2022; Xu et al., 2021a) have been employed to harmonize datasets across experimental replicates or conditions. By correcting these confounding factors, computational methods ensure that observed perturbation effects are consistent and biologically relevant, improving the reliability of downstream analyses.

Emerging tools are increasingly designed to predict the outcomes of perturbations before they are experimentally tested. Predictive models, powered by artificial intelligence and trained on existing perturbation datasets, are now being used to anticipate how specific genetic edits affect cellular states. This capability accelerates hypothesis testing and enables researchers to design more efficient and focused perturbation screens. For example, recent studies (Kamimoto et al., 2023; Lotfollahi et al., 2019; Zhan et al., 2024) have applied deep learning to generate virtual perturbation profiles, offering a cost-effective and scalable way to explore potential gene functions and interactions.

As single-cell perturbation technologies continue to evolve, the computational ecosystem must also adapt to keep pace with the growing complexity of data and experimental design. The ongoing development of integrative, scalable, and predictive tools promises to expand the utility of single-cell CRISPR screens, deepening our understanding of cellular processes and enabling discoveries in both basic and translational research.

## 5 Application of CRISPR genome editing in cancer and immunology

### 5.1 Cancer therapy using genome editing

CRISPR-based gene editing technologies are revolutionizing cancer therapy by enabling precise genomic modifications, enhancing treatment efficacy, and introducing novel therapeutic strategies. These advancements hold immense potential for improving targeted interventions and overcoming current limitations in cancer treatment.

#### 5.1.1 *Ex vivo* CRISPR therapies

**FIGURE 5:**
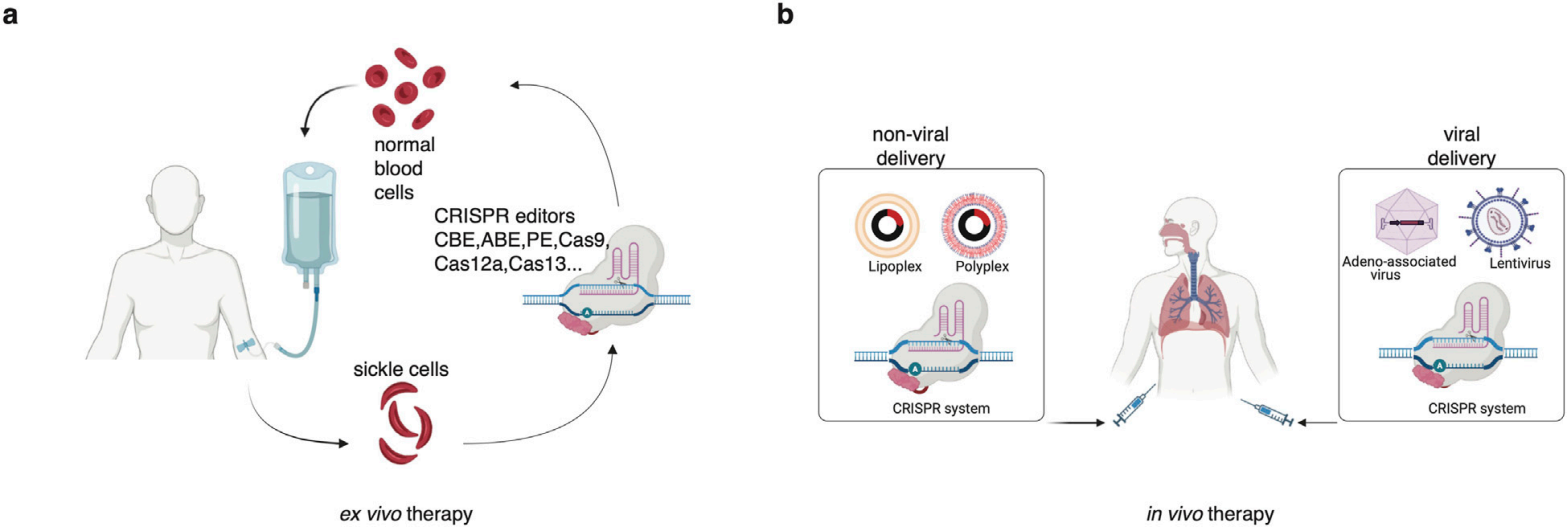
Outline of *ex vivo* **(a)** and *in vivo* **(b)** treatment of genetic disease using CRISPR-Cas system.

*Ex vivo* CRISPR therapies leverage the genome-editing capabilities of CRISPR-Cas systems to reprogram cells outside the patient’s body before reinfusion. This approach has demonstrated significant promise in augmenting the efficacy of immunotherapies, particularly in immune checkpoint inhibition and adoptive cell therapy (Figure 5A).

CRISPR-Cas9 has notably advanced adoptive T cell therapies, particularly in enhancing Chimeric Antigen Receptor (CAR) T cell treatments (Zhou X. et al., 2023). While traditional CAR-T therapies have shown success in treating hematological malignancies, several challenges persist, including limited persistence, graft-versus-host disease (GVHD), cytokine release syndrome (CRS), off-target effects, and inefficiencies in autologous cell production (Sterner and Sterner, 2021). CRISPR addresses these challenges by enabling precise gene modifications that enhance T cell function and longevity. Multiple studies have demonstrated that *ex vivo* CRISPR-based disruption of PD-1 in T cells enhances anti-tumor activity post-adoptive transfer (Guo et al., 2018; Nakazawa et al., 2020; Zhao et al., 2018). Notably, a more efficient and flexible precision gene editing platform named CLASH (Cas12a/Cpf1-based Library-scale AAV-perturbation with Simultaneous HDR-knockin) was recently developed to facilitate massively parallel knock-in engineering in human cells, which enables high-throughput engineering of CAR-T cells and simultaneously identifies the best candidates for potential therapeutic applications (Dai et al., 2023). As of 2025, over 30 clinical trials have been registered for CRISPR-engineered T cells in cancer treatment, highlighting the expanding clinical interest in this transformative technology. For example, CRISPR has facilitated the development of allogeneic CAR-T cells by knocking out genes responsible for GVHD and immune rejection, paving the way for universally compatible, off-the-shelf CAR-T therapies that increase patient accessibility (Hu et al., 2022). Ottaviano et al. used CRISPR-Cas9 editing to disrupt T cell receptor α chain and remove CD52 in CAR19 T cells to create a universal cell therapy, which met safety goals in a phase 1 trial for CD19^+^ B-ALL, highlighting CRISPR’s therapeutic potential (Ottaviano et al., 2022).

Natural killer (NK) cells present an alternative to T cells for CAR therapy, avoiding severe GVHD or CRS. Recent studies indicate that CAR-NK cells represent a potent cancer therapy option, particularly in allogeneic settings, thereby reducing costs and increasing accessibility (Liu E. et al., 2020; Xiao L. et al., 2019). CRISPR has been employed to knock out genes that inhibit CAR expression or to optimize receptor design, thereby enhancing NK cell affinity and specificity for tumor antigens. For example, CRISPR-mediated knockout of PD-1 has significantly improved NK cell activity, cytokine production, and tumor cell cytotoxicity (Pomeroy et al., 2020). In contrast, disruption of the NKG2A-encoding killer cell lectin-like receptor C1 (KLRC1) has enhanced NK cell-mediated anti-tumor responses against multiple myeloma (Bexte et al., 2022). In Phase I/II clinical trials, allogeneic CD19-targeted CAR-NK cells demonstrated promising response rates in patients with B-cell malignancies while maintaining an excellent safety profile, with no reported cases of severe cytokine release syndrome, neurotoxicity, or graft-versus-host disease (Marin et al., 2024; Liu E. et al., 2020).

#### 5.1.2 *In vivo* CRISPR therapies

*In vivo*, CRISPR-based cancer therapies involve direct gene editing within living organisms to target oncogenes (e.g., KRAS, c-Myc, Figure 5B) and tumor suppressor genes (e.g., p53), as well as to modulate immune cells such as T cells and NK cells, offering the potential to target and modify cancer within the body directly. Currently, clinical trials specifically employing *in vivo* gene editing for cancer treatment are limited. However, the field is rapidly evolving, with ongoing research focused on refining delivery methods and ensuring the safety and efficacy of these therapies. It is important to note that while *in vivo* gene-editing therapies for cancer are still in the early stages, the promising results from preclinical studies and advancements in related fields, such as rare diseases, suggest potential for future clinical applications.

*In vivo*, CRISPR therapies require efficient delivery systems to transport CRISPR components into target cells within the body. These methods can be broadly categorized into **viral** and **non-viral** approaches. The choice of different methods impacts the efficacy, specificity, safety, and clinical feasibility (Supplementary Table 5). Adeno-associated viruses (AAVs) and lentiviruses are the most studied viral vectors for delivering CRISPR-Cas9 systems *in vivo*. While these vectors can be engineered for targeted tumor delivery, their limited cargo capacity and immunogenicity pose constraints. Conversely, lipid nanoparticles (LNPs) have gained traction for nucleic acid delivery. For instance, LNPs encapsulating Cas9 mRNA and gRNAs targeting polo-like kinase 1 (PLK1) in a glioblastoma mouse model demonstrated effective gene editing and tumor suppression (Rosenblum et al., 2020).

To mitigate off-target effects, high-fidelity Cas enzymes such as HF1 (Kleinstiver et al., 2016), HiFi (Vakulskas et al., 2018), and HypaCas9 (Ikeda et al., 2019), along with improved sgRNA designs have been developed. Base editing, which enables precise nucleotide modifications without inducing double-strand breaks, further reduces off-target risks. A landmark clinical application of base editing involved treating a 13-year-old girl with T-cell acute lymphoblastic leukemia, resulting in remission—marking base editing as a promising alternative to traditional CRISPR editing. However, any genome manipulation has potential functional consequences that require thorough evaluation before clinical implementation.

#### 5.1.3 CRISPR screen strategies in cancer immunology

CRISPR-based gene editing and functional genetic screens enable the systematic identification of genes and pathways that regulate immune-cancer interactions, thereby facilitating the discovery of novel therapeutic targets. These screens typically apply selective pressures, including cytotoxic T cells, immune checkpoint inhibitors, cytokines, or co-culture systems that mimic immune-tumor interactions (Lizotte et al., 2018). Both *in vitro* and *in vivo* CRISPR screens have been successfully developed to identify novel immunotherapy targets (Parnas et al., 2015; Schumann et al., 2020; Shifrut et al., 2018; Su et al., 2024; Wei et al., 2019; Ye et al., 2019). For example, genome-wide CRISPR screens in primary human T cells have elucidated critical components of T cell receptor signaling, facilitating the identification of functional genetic targets in immune cells (Shifrut et al., 2018). In a murine T cell line, a genome-wide knockout screen identified FUT8 as a regulator of PD-1 surface expression (Okada et al., 2017). An *in vivo* genome-scale CRISPR screen was performed in CD8 T cells directly under cancer immunotherapy settings and identified novel regulators of T cell tumor infiltration and degranulation, such as a previously uncharacterized helicase, DHX37 (Dong et al., 2019). Similarly, CRISPR screens in xenograft models identified MEN1 as a dual regulator of tumor–microenvironment interactions (Su et al., 2024). In solid tumor mouse models, *in vivo* screens of tumor-infiltrating NK cells revealed endogenous genetic checkpoints limiting NK cell function, highlighting the role of CALHM2 knockout in enhancing NK cell-based immunotherapies (Peng et al., 2024). Furthermore, integrating CRISPR screens with single-cell RNA sequencing (scRNA-seq) has enabled the identification of key transcriptional regulators in human regulatory T (Treg) cells, uncovering potential immunotherapeutic targets (Schumann et al., 2020).

As research continues to unveil novel targets regulating anti-tumor immune responses (Zhang et al., 2025), CRISPR screening methodologies provide invaluable mechanistic insights that significantly expand the landscape of cancer immunotherapy.

### 5.2 Autoimmune therapy by genome editing

CRISPR technology has been diversified to treat autoimmune disorders by modulating dysregulated immune responses. Recent studies have identified potential target genes for immunomodulation in various autoimmune diseases, including rheumatoid arthritis, inflammatory bowel diseases, systemic lupus erythematosus (SLE), multiple sclerosis (MS), type 1 diabetes mellitus (T1DM), psoriasis, and type 1 coeliac disease (Chehelgerdi et al., 2024; Lee et al., 2022; Ozanne et al., 2000). Notably, clinical trials employing CRISPR-modified T cells successfully knocked out genes implicated in immune rejection, achieving complete B-cell depletion and ameliorating pathological conditions in patients (Wang et al., 2024).

Innovative approaches combining CRISPR-Cas9 with transcription activator-like effector nucleases (TALENs) have demonstrated enhanced specificity and efficiency in editing immune-regulatory genes, with significant success in treating autoimmune conditions such as SLE and MS (Ozanne et al., 2000). This hybrid strategy leverages the strengths of both systems, enabling precise engineering of immune cells to resist autoimmunity. Mechanistically, these systems achieve therapeutic efficacy by either knocking out autoreactive genes or inserting regulatory elements to suppress pathological immune responses, thereby mitigating disease progression and improving patient outcomes.

Similarly, CRISPR-Cas12a has advanced therapeutic applications in autoimmune disorders by targeting immune-related genes with high specificity (Houghton et al., 2022). In Type 1 Diabetes Mellitus (DM), CRISPR-Cas9 has confirmed and edited single nucleotide polymorphisms (SNPs) directly associated with disease progression, offering new avenues for intervention (Zhu et al., 2019). Additionally, studies have identified the critical role of ERAP1 genes in Psoriasis, where gene knockout significantly reduced disease progression (Arakawa et al., 2019).

Beyond direct therapeutic applications, CRISPR-based technologies have facilitated the development of disease models replicating specific gene mutations implicated in autoimmunity (Xu and Li, 2020). These models provide invaluable insights into the pathogenesis of autoimmune disorders, enabling researchers to dissect disease mechanisms and identify novel therapeutic targets. For example, CRISPR-engineered models have elucidated critical pathways in diseases such as SLE and MS, guiding the development of precision medicine approaches.

In conclusion, the advancements in CRISPR technologies hold the potential for transformative therapies in autoimmune diseases and pave the way for more accurate disease modeling. These innovations deepen our understanding of autoimmune pathophysiology and open new frontiers for precision medicine, ultimately improving outcomes for patients with these complex disorders.

### 5.3 Genome editing in diagnosing and treatment of infectious disease

CRISPR technology has significantly advanced diagnostics for infectious diseases caused by pathogens such as dengue (Li et al., 2020), Zika (Dukhovny et al., 2019), HIV (Xiao Q. et al., 2019), and Tuberculosis (Zein-Eddine et al., 2023). Tools like DETECTR (Broughton et al., 2020) and SHERLOCK (Kellner et al., 2019) leverage CRISPR’s specificity to detect viral RNA or DNA with exceptional sensitivity, providing rapid, user-friendly, and portable diagnostic solutions suitable for point-of-care testing (Lou et al., 2022). Additionally, innovative CRISPR-based detection platforms, such as HUDSON, have demonstrated the capability to detect infections directly from patient samples, expanding the utility of CRISPR diagnostics in clinical settings.

Beyond diagnostics, CRISPR is utilized to combat infectious diseases by developing antimicrobial agents that selectively target specific pathogens based on their genetic sequences (Bikard et al., 2014). Successful studies on *Staphylococcus aureus* (Diep et al., 2006; Weigel et al., 2003) have demonstrated the potential of CRISPR-based treatments for antibiotic-resistant infections. CRISPR also plays a pivotal role in vaccine development, identifying novel antigen candidates (Wang and Doudna, 2023) and inhibiting viral replication (Komissarov et al., 2022). Recent advancements have led to the development of vaccines for infections such as H5N1, DTMUV, and AIV, showcasing the growing relevance of CRISPR in preventive healthcare and treating infectious diseases (Chang et al., 2019; Zou et al., 2017).

## 6 Conclusion and discussion

The review highlights the importance of CRISPR genome editing, single-cell technologies, and artificial intelligence (AI) in advancing precision medicine, therapeutics, and functional genomics. The convergence of these technologies has redefined our ability to interrogate cellular heterogeneity, gene regulation, and disease mechanisms with unprecedented precision. The synergy between CRISPR and single-cell platforms like **Perturb-seq** (Dixit et al., 2016), **CROP-seq** (Datlinger et al., 2017), and **CRISP-seq** (Jaitin et al., 2016) has been instrumental in advancing cancer and immunology research by facilitating the identification of key regulators of tumor progression, immune dynamics, and resistance mechanisms. Furthermore, multi-omics platforms such as **MultiPetrub-seq** (Metzner et al., 2025), **Spear-ATAC** (Pierce et al., 2021), **Petrub-ATAC** (Rubin et al., 2019), and **CRISPR-sciATAC** (Liscovitch-Brauer et al., 2021) have expanded our understanding of genetic perturbations by integrating single-cell transcriptomics and chromatin accessibility, enabling a deeper analysis of cellular regulatory networks. AI-driven approaches have refined these methodologies by enhancing sgRNA design precision, optimizing genome editing workflows, and improving multi-omics data analytics. Deep learning (Kim et al., 2019) and transformers (Zhou Z. et al., 2023) based models have facilitated the identification of novel biomarkers, modeled tumor evolution, and personalized therapeutic strategies, demonstrating AI’s essential role in leveraging CRISPR and single-cell technologies for translational research and clinical applications.

Despite these significant advancements, several challenges remain, particularly in ensuring the reproducibility of single-cell CRISPR screens. Single-cell technologies inherently suffer from technical variability, including batch effects, dropout events, and noise in transcriptomic data, which can affect the reliability of perturbation outcomes. The efficiency of CRISPR-based perturbations is also subject to variability in guide RNA delivery, editing efficiency, and transcriptional responses across different experimental conditions. Standardizing experimental protocols, including improved guide RNA design algorithms, robust single-cell data preprocessing pipelines, and developing high-throughput benchmark datasets, is crucial to addressing these issues. Furthermore, computational frameworks for integrating single-cell CRISPR datasets across multiple studies and experimental conditions require enhanced batch correction and normalization strategies. AI-driven harmonization techniques, such as deep generative models, variational autoencoders, and transfer learning approaches, hold promise for improving reproducibility in single-cell CRISPR perturbation screens. Expanding multi-omic approaches to include proteomics, metabolomics, and spatial omics will further enhance our understanding of cellular functions and necessitate innovative computational solutions to harness these modalities fully. Standardizing AI-driven methods across diverse datasets is essential to ensuring their robustness and broad applicability.

The delivery systems for CRISPR therapeutics remain a significant bottleneck, as current approaches rely heavily on viral (Sioson et al., 2021) or lipid-based nanoparticles, both of which have limitations in efficiency, specificity, and scalability. Developing non-viral delivery systems, tissue-specific targeting mechanisms, and minimally invasive administration techniques is crucial for broadening the clinical applicability of CRISPR technologies. Advances in nanoparticle engineering, RNA-based delivery systems, and biomaterial-based carriers may provide novel solutions to enhance the precision and safety of CRISPR-based therapies. Additionally, off-target effects (Zhang et al., 2015) and delivery efficiency (Sioson et al., 2021) remain key challenges that must be addressed to improve genome editing technologies’ specificity and therapeutic potential.

Beyond technical and biological challenges, the equitable distribution of these advanced therapies poses a socio-economic barrier to global implementation. While CRISPR and AI-driven approaches hold promise for personalized medicine, disparities in access to these innovations persist, particularly in resource-limited settings. The integration of CRISPR with multi-omics technologies offers the potential for personalized immunotherapies tailored to individual genetic profiles; however, the ethical considerations surrounding genome editing and the regulatory complexities of clinical translation require careful oversight. Global regulatory frameworks are needed to ensure accessibility across diverse populations and minimize disparities in healthcare innovation.

Integrating CRISPR, single-cell platforms, and AI-driven models can revolutionize basic and translational research by addressing these challenges and fostering interdisciplinary collaboration. As these technologies evolve, they promise to advance human health by providing transformative solutions to decode biological complexity and translate genomic insights into effective clinical therapies.
